# Essential role for Argonaute2 protein in mouse oogenesis

**DOI:** 10.1186/1756-8935-2-9

**Published:** 2009-08-10

**Authors:** Masahiro Kaneda, Fuchou Tang, Dónal O'Carroll, Kaiqin Lao, M Azim Surani

**Affiliations:** 1 Wellcome Trust Gibbs Building 215 Euston Road London NW1 2BE, UK; 2 Cancer Research UK Gurdon Institute of Cancer and Developmental Biology, University of Cambridge, Cambridge, UK; 3 The Laboratory for Lymphocyte Signaling and the Laboratory of Molecular Immunology, The Rockefeller University, New York, USA; 4 European Molecular Biology Laboratory, Mouse Biology Unit, Monterotondo Scalo, Rome, Italy; 5 Molecular Cell Biology, Applied Biosystems, Foster City, CA, USA; 6 Reproductive Biology and Technology Research Team, National Institute of Livestock and Grassland Science, National Agriculture and Food Research Organization, Tsukuba, Japan

## Abstract

**Background:**

Argonaute2 protein (Ago2) is a key component of RNA-induced gene silencing complex, which is crucial for microRNA-mediated repression of target genes. The function of Ago2 in the mouse oocyte and early embryonic development is less well characterized but it is likely to have an important role in regulating maternally inherited mRNA. We have examined the role of Ago2 by conditional deletion of the gene in developing oocytes.

**Results:**

Ago2 was deleted specifically in the growing oocytes. Although the Ago2-deficient oocytes are able to develop to mature oocytes, they have abnormal spindles and chromosomes that are unable to cluster together properly. This phenotype is very similar to the phenotype of Dicer-deficient oocytes. We examined the microRNA expression profile in the Ago2-deficient oocyte and found that the expression of most microRNAs was reduced by more than 80%. To determine the downstream genes that are regulated by Ago2, we used microarray analysis on Ago2-deficient oocytes and found that 512 genes were upregulated and 1,073 genes were downregulated (FC > 2, *P *< 0.05).

**Conclusion:**

Our study shows that Ago2 has a key function in the mouse oocyte through global regulation of microRNA stability, and through this mechanism it affects gene expression in developing oocytes.

## Background

MicroRNAs (miRNAs) are single-stranded RNA molecules of around 21 to 23 nucleotides that play a variety of roles in gene expression regulation in different species [1-5]. We and others have previously shown that Dicer, an RNase III family ribonuclease that cleaves miRNA precursor (pre-miRNA) into mature miRNA, is essential for mouse oogenesis [6,7]. Dicer-deficient oocytes were almost entirely depleted of miRNAs and showed disorganized spindle formation. Mature miRNAs are loaded into an RNA-induced gene silencing complex (RISC) that contains Argonaute (Ago) family proteins [8]. All four members, Ago1 to Ago4, can repress target gene expression through either the reduction in the length of the polyA tail of mRNA, or by inhibiting translation [9]. However, only Ago2 has the slicer activity that cuts the targeted mRNA recognized by miRNAs. Here we describe the effect of loss of Ago2 in mouse oogenesis by specific deletion of Ago2 in the growing oocyte.

## Results

### Phenotype of Ago2-deficient oocytes

It is known that loss of function of Ago2 leads to embryonic lethality at around embryonic day 9.5 (E9.5) [8]. However, the role of Ago2 during oogenesis and in early embryonic development is unknown. To understand the function of Ago2 in mouse oogenesis and early embryonic development, we generated [Ago2^F/F^, Zp3-Cre] mice to disrupt Ago2 function specifically in growing oocytes by deletion of the Ago2 floxed conditional allele [9], by crossing with Zp3-Cre transgenic mice that express Cre recombinase under the control of zona pellucida glycoprotein 3 promoter (Zp3) in the growing oocyte [10]. It is known that Zp3 is expressed only in the growing oocytes after birth. Thus, this allows us to knockout Ago2 exclusively in the growing oocytes in order to examine the role of Ago2 in oogenesis and early development.

**Figure 1:**
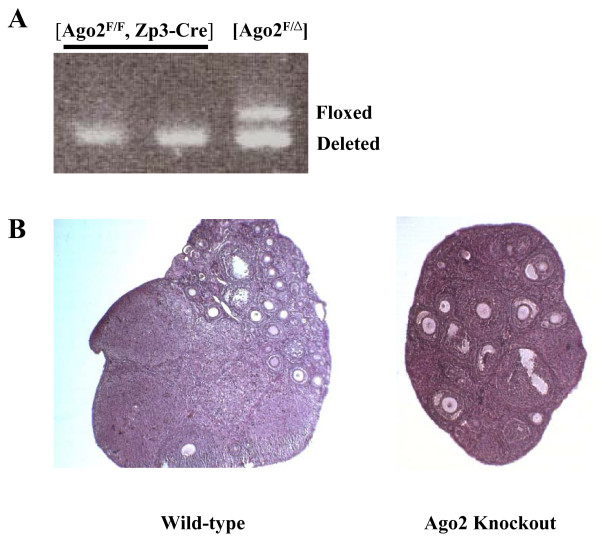
**Genotyping of oocytes isolated from P10 (ten days after birth) Ago2 conditional knockout females**. **(A) **Both floxed and deleted Ago2 alleles are amplified simultaneously by specific primer pairs. **(B) **HE staining of ovaries from Ago2 conditional knockout and wild-type females shows a number of fully grown oocytes in both ovaries.

**Figure 2:**
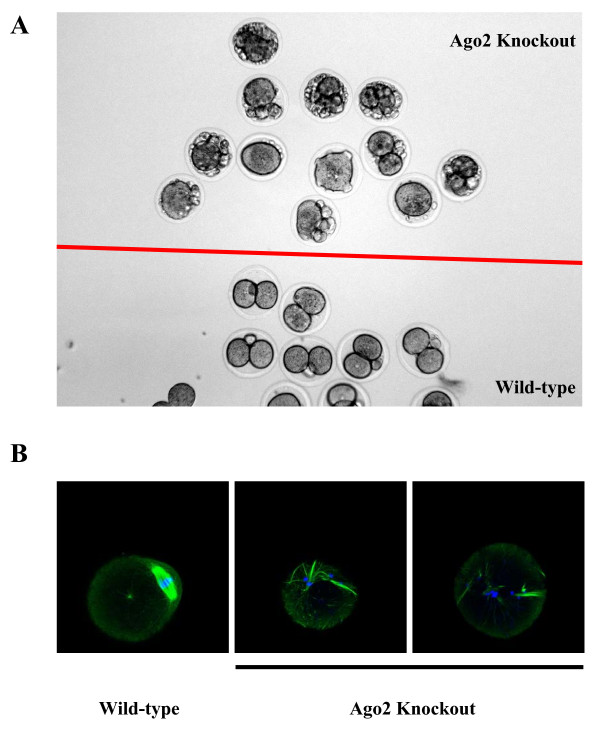
**Morphology of E1.5 embryos derived from Ago2-deficient oocytes and wild-type oocytes**. **(A) **Embryos above the red line are Ago2 knockout, while embryos below the red line are wild-type controls. Spindle formation in wild-type (left) and Ago2-deficient (right) ovulated metaphase II oocytes. **(B) **The spindle was stained with rat monoclonal anti-tubulin (YL 1/2, Abcam) antibody (Green) and DNA was counterstained with DAPI (blue).

First, we isolated oocytes from postnatal day 10 (P10) conditional knockout females to examine the efficiency of deletion by Zp3-Cre and found that almost all the oocytes had already undergone Cre-mediated deletion at this time (Figure 1A). We then mated these conditional knockout females with wild-type males to test for development of fertilized Ago2-depleted oocytes. None of the females were found to be pregnant from these crosses although the ovaries looked normal (Figure 1B), and the ovulated metaphase II (MII) oocytes were morphologically indistinguishable from wild-type oocytes in their appearance, maturity, size and number (data not shown). When we recovered early embryos at E1.5 from mutant and control females mated with the wild-type males, we found that most of the mutant embryos had failed to undergo the first cleavage division and appeared to be fragmented (Figure 2A and Table 1). This was also the case at E2.5. There were rare exceptions, however, where a few embryos looked normal at E1.5, and these developed to normal blastocyst stage *in vitro *(Table 1). We isolated genomic DNA from these embryos for genotyping and found that they contained an intact Ago2 allele, which indicates that in a few cases the floxed Ago2 allele fails to be deleted by Zp3-Cre during oogenesis (data not shown).

**Table 1:** Number of E1.5 and E2.5 embryos derived from wild-type (WT) and Ago2-deficient (KO) oocytes.

	Litters	Non-divided	2-cell	8-cell	Fragmented
E1.5 (WT)	5	1	44 (83%)	-	8

E1.5 (KO)	21	87	9 (5%)	-	80

E2.5 (WT)	2	0	0	12 (100%)	0

E2.5 (KO)	2	2	0	0 (0%)	8

Embryos were isolated from the oviducts of wild-type and Ago2 conditional knockout females one day (E1.5) or two days (E2.5) after plugged by wild-type males.

To understand the mechanism of the developmental arrest phenotype, we checked the spindle organization of these Ago2-deficient oocytes by fluorescent immunostaining for tubulin. We found that the spindle formation is abnormal in knockout oocytes, and the chromosomes did not cluster together properly (Figure 2B). So although the morphology of the oocytes is totally normal, these Ago2-deficient oocytes already have a severe defect of spindle formation and chromosome arrangement, which is similar to the defect observed with the Dicer knockout oocytes [6].

### Reduced miRNA expression and disruption of gene expression pattern in Ago2-deficient oocytes

**Figure 3:**
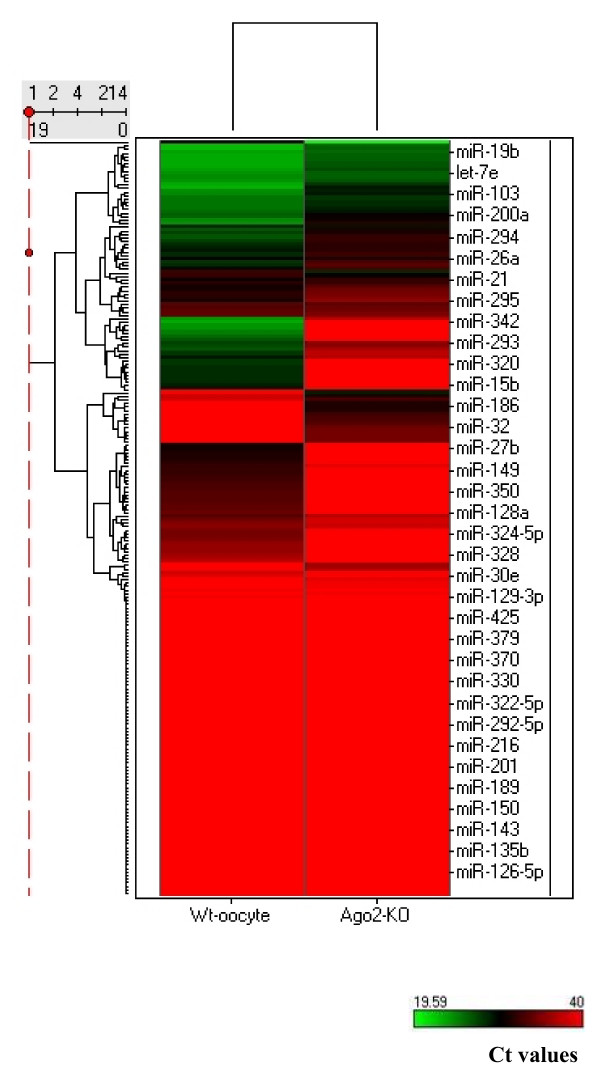
**Hierarchical clustering heat map of 220-plex miRNA expression profiles**. 220-plex miRNA expression profiles between wild-type (Wt) and Ago2-KO oocytes were shown as a heat map based on the Ct value of each miRNA.

Argonaute proteins are important for mediating the function of miRNAs. To further understand the function of Ago2 in relation to miRNAs, we carried out real-time PCR-based miRNA profiling to compare Ago2-deficient mature oocytes with wild-type controls [11,12]. We found that there is a decrease in miRNAs of more than 80% (Figure 3 and Table 2), which indicates that Ago2 is crucial for the biogenesis or stability of miRNAs during oogenesis.

**Table 2:** The relative expression levels of let-7 family miRNAs in wild-type (WT) and Ago2-deficient (KO) oocytes.

	WT	KO	KO/WT
let-7a	9,034	9	0.1%

let-7b	4,517	246	5.4%

let-7c	5,189	246	4.7%

let-7d	2,421	115	4.8%

let-7e	6,388	399	6.2%

let-7f	6,847	399	5.8%

let-7g	2,594	81	3.1%

let-7i	2	0	0.0%

The relative amount of each miRNA was determined by using standard curve of synthesized miR-16.

To further understand the downstream genes regulated by Ago2, we performed single cell cDNA microarrays and compared Ago2-deficient oocytes with the wild-type controls [6,13]. We found that 512 genes are upregulated and 1,073 genes are downregulated in Ago2-deficient oocytes (FC > 2, *P *< 0.05; Additional file 1). We confirmed the array results by real-time PCR (Additional file 2). This suggests that a large proportion of genes in oocytes are regulated by Ago2.

Previously we showed that Dicer is essential for oogenesis, and the phenotypes of Dicer- and Ago2-deficient oocytes are remarkably similar [6,7]. We therefore asked if the downstream genes affected by these two knockouts are also similar. We compared genes affected by these two knockouts and found that, in fact, only a proportion of the genes regulated by Ago2 and Dicer overlap (Additional file 1). Dicer and Ago2 regulate a large number of genes but they appear to be distinct sets of genes [6]. This proves that although the phenotype of the Dicer- and Ago2-deficient oocyte is similar, the downstream genes affected are distinct.

**Figure 4:**
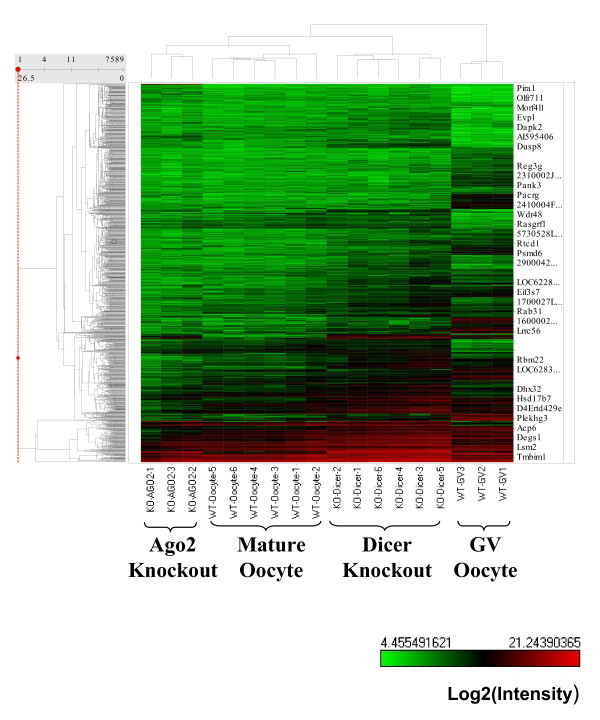
**Heat map of single cell cDNA microarray of oocytes**. Unsupervised hierarchical structure heat map of single cell cDNA microarray of wild-type (WT) GV oocyte, wild-type mature oocyte, Ago2-deficient mature oocyte, and Dicer-deficient mature oocyte. All 7,589 *P *< 0.01 genes were shown.

We next examined the microarray results in more detail by asking how the Ago2 knockout affects genes that are regulated dynamically from the germinal vesicle (GV) oocyte stage to mature oocyte stage. We found that for the genes upregulated from GV oocyte to mature oocyte (1,470 genes), 39% (572 genes) are downregulated in Ago2 mutants (Figure 4) whereas only 5% of them (69 genes) are downregulated in the case of Dicer knockout [6]. This indicates that Ago2 is more important for turning on mature oocyte specific genes during development from GV oocyte stage to mature oocyte stage. On the other hand, of genes downregulated from GV oocyte to mature oocyte stage (5,090 genes), 10% (486 genes) are upregulated in Ago2 mutants (Figure 4), whereas 52% of them (2,640 genes) are upregulated in Dicer mutants [6]. This indicates that Dicer is more important for clearance of GV oocyte-specific genes during development from GV oocyte stage to mature oocyte stage. This also indicates that Ago2 and Dicer have distinct roles. Further analysis of these mutants may reveal the role of miRNAs in mouse oogenesis.

## Discussion

Argonaute2 protein is the core component of RISC. This protein binds to miRNAs which, in turn, mediates repression of their target mRNAs through post-transcriptional gene silencing. Ago2 is a unique member of Argonaute protein family because it has slicer activity that can cut perfect complement targets of miRNAs and small interfering RNAs (siRNAs) [8]. It has recently been shown that Ago2 also has an important slicer-independent activity for miRNA processing and/or stability [9]. Until this study, however, the function of Ago2 in mouse oogenesis and early embryonic development was unknown. By deleting Ago2 specifically in the growing oocyte by mating with Zp3-Cre mice, we found that these females are sterile. Although Ago2-deficient oocytes can develop to morphologically normal mature oocytes, the spindle and the organization of the chromosomes are abnormal. We analyzed the miRNA expression profile in the Ago2-deficient oocyte and found that miRNAs were globally downregulated by over 80%. This showed that Ago2 is crucial for stabilizing and/or processing of miRNAs in oocytes. We also analyzed the downstream genes regulated by Ago2 by single cell microarray in wild-type and Ago2-deficient oocytes. There was a large-scale change in the gene expression profile after deletion of Ago2. The genes affected by Ago2 knockout only partially overlap with the genes affected by Dicer knockout, which means that although the phenotype is similar, the oocyte-specific Ago2 knockout shown here represents a unique model for post-transcriptional gene silencing in oocytes.

These results contrast with the recent publication from our laboratory on development of Dicer- and Ago2-deficient primordial germ cells (PGCs) and spermatogonia [14]. Dicer-deficient PGCs and spermatogonia showed poor proliferation, whereas spermatogenesis in Ago2-deficient testis was normal. It is possible that there are distinct roles for miRNAs in oogenesis and spermatogenesis, especially considering that there are a lot of Piwi-interacting RNAs (piRNAs) in mouse testis [15-19]. However, more recently, two groups demonstrated that there are a number of endogenous double-stranded RNAs (dsRNAs) in mouse oocytes and that they control retrotransposon expression and protein-coding transcripts by forming dsRNA structures [20,21]. piRNAs are also expressed in mouse oocytes and bound by MILI, which regulates retrotransposon expression [20]. Furthermore, Dicer- and Ago2-deficient oocytes showed decreased siRNA expression and increased mRNA expression complementary to the siRNAs [20,21]. Retrotransposons are also de-repressed in the mutant oocytes [20]. These results indicate diverse roles for Dicer and Ago2 in mouse oocytes; they are not only involved in the miRNA pathway, but also drive the siRNA pathway and control gene expression via RNAi machinery.

Morita *et al*. reported results from another line of Ago2 knockout mice that showed a more severe phenotype, dying at around E6.5, which is very similar to Dicer knockout mice [22,23]. Their knockout mice are a gene-trap line, which disrupts exon 1 instead of exons 9 to 11 in our knockout mice shown here. It is possible that their knockout is a null mutant, while ours may be a hypomorph. Nevertheless, de-repression of retrotransposons was not observed in knockout embryos, whereas the retrotransposons MT and RLTR10 were highly expressed in our Ago2-deficient oocytes [20]. This difference can be explained because MT and RLTR10 are mouse oocyte-specific retrotransposons, so there could be different regulation of retrotransposon expression between somatic cells and oocytes.

## Conclusion

Our studies reveal that Ago2 is essential for mouse oogenesis but apparently not for spermatogenesis [14]. Ago2 also has a role in miRNA homeostasis, suggesting a specific role for Ago2 in RISC. Our results, together with those from Watanabe *et al*. and Tam *et al*. [20,21] suggest that siRNAs, as well as miRNAs, are regulated by Dicer and Ago2 in the mouse oocyte to control retrotransposon expression and mRNA expression. Our work may help to pave the way to understanding of the molecular mechanism of miRNA/siRNA genesis and functions, and related processing machinery.

## Methods

### Mice and oocytes

Work on mice described in this paper has ethical approval from the University of Cambridge and has been carried out under an animal project license issued by the UK Home Office to MAS. The Ago2 floxed mice were mated with Zp3-Cre transgenic mice to obtain [Ago2^F/F^, Zp3-Cre] conditional knockout females [9,10]. Mice were genotyped by polymerase chain reaction (PCR; primers sequences and PCR conditions are available from the authors on request). Growing oocytes were obtained from the ovaries of P10 females, according to the protocol described previously [24]. Ovulated MII oocytes were collected from the oviducts from adult females and treated with hyaluronidase to remove the cumulus cells.

### miRNA expression analysis

miRNA expression from each oocyte was examined by a protocol described previously [11,12]. Briefly, individual oocytes were lysed at 95°C for 5 min and then all miRNAs were reverse transcribed in a 5 μl reaction. cDNAs were then amplified by pre-PCR in a 25 μl reaction. After the amplification, all miRNAs were assayed individually by TaqMan probe-directed real-time PCR by ABI 7000 system [12]. The relative amount of miRNAs was deduced based on the standard curve of synthesized miR-16 mature miRNA under exactly the same conditions. All primers and probes used were listed previously (see supplement of [11]).

### Microarray analysis

Samples underwent *in vitro *transcription (IVT) to synthesize DIG-labeled cRNA using part of the Applied Biosystems NanoAmp RT-IVT protocol (Section 2 onwards of protocol WISe_0025_v01). All cDNA samples were normalized to 10 ng/μl and 60 ng was used in IVT (see worksheet RNW024-01). Samples were then QC'd for quantity (nanodrop spectrophotometer) and quality (Agilent Bioanalyzer). 10 μg of cRNA was fragmented and subsequently prepared for hybridization to Applied Biosystems V2.0 Mouse Genome Microarray in accordance with The Applied Biosystems Chemiluminescence Detection Protocol (WISe_0018_v02) [25]. Following hybridization, arrays were stained, washed, and finally scanned using the Applied Biosystems 1700 Chemiluminescent Microarray Analyzer.

### Immunostaining

Oocytes were fixed in 4% paraformaldehyde/PBS for 10 min, and washed with PBS three times. After 1 h incubation with blocking buffer (1% bovine serum albumin and 0.1% Tween-20 in TBS), oocytes were incubated overnight with the anti-tubulin antibody (YL 1/2, Abcam, UK) diluted with blocking buffer to 1:100 at 4°C. After extensive washes, the oocytes were incubated for 30 min with an appropriate Alexa488-conjugated second antibody diluted to 1:100 at room temperature. After washing with PBS three times, DNA was counterstained with DAPI.

### Accession numbers

All the data of the single cell cDNA microarray were deposited in GeneBank [26]. The accession number is GSE15529.

## Competing interests

The authors declare that they have no competing interests.

## Authors' contributions

MK, FT and MAS conceived of and designed the experiments. MK, FT and KL performed the experiments. MK, FT, KL and MAS analysed the data. DO generated conditional knockout mice. MK, FT, KL and MAS wrote the paper. All authors read and approved the final manuscript.

## Supplementary Material

Additional file 1 **Heat map of wild type (WT), Ago2-deficient (Ago2) and Dicer-deficient (Dicer) oocyte cDNA microarray from single oocyte**. Genes that are differentially expressed between wild-type (left six columns), Ago2-deficient (middle three columns) and Dicer-deficient oocytes (right six columns) are shown (*P *values (FC(AGO2-KO/Wt-oocyte)) < 0.05 and FC (AGO2-KO/Wt-oocyte) >2 or <0.5). Click here for file

Additional file 2 **Real-time PCR measurement of candidate gene expression**. Seven genes showing differential expression based on array results were checked by single cell cDNA real-time PCR. Six of them were confirmed as showing differential expression. Click here for file
