# Supplementary figures and images for: Essential role for Argonaute2 protein in mouse oogenesis
Source: Epigenetics Chromatin. 2009 Aug 10;2:9. doi: 10.1186/1756-8935-2-9 (PMC2736168; doi:10.1186/1756-8935-2-9)

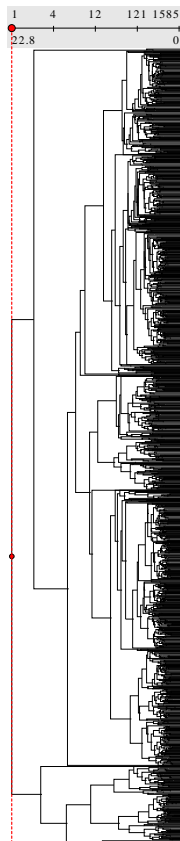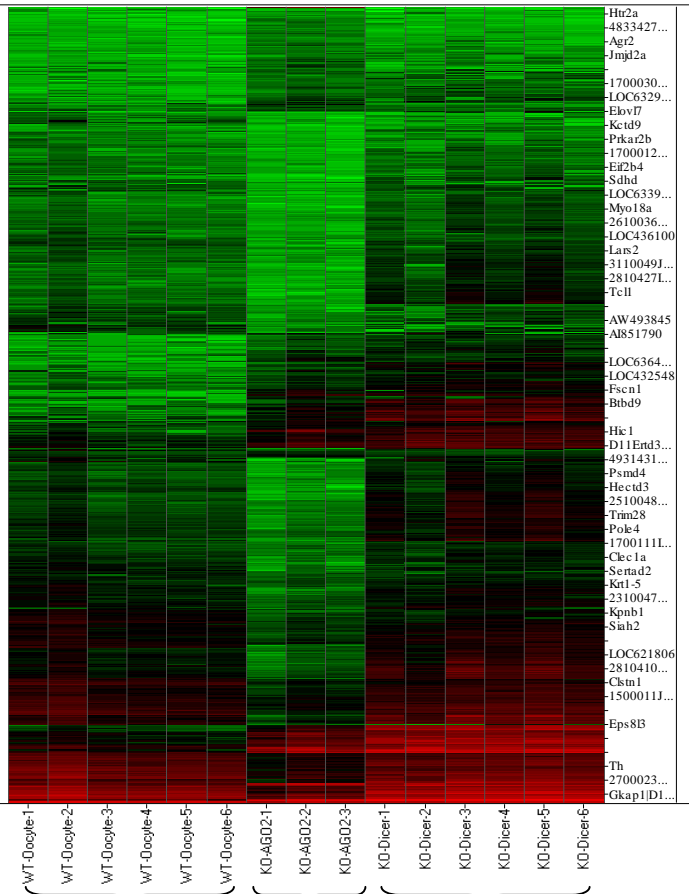

**Wild-type  
Oocyte**

**Ago2  
Knockout**

**Dicer  
Knockout**

Supplement: Additional file 1 — Heat map of wild type (WT), Ago2-deficient (Ago2) and Dicer-deficient (Dicer) oocyte cDNA microarray from single oocyte. Genes that are differentially expressed between wild-type (left six columns), Ago2-deficient (middle three columns) and Dicer-deficient oocytes (right six columns) are shown (P values (FC(AGO2-KO/Wt-oocyte)) < 0.05 and FC (AGO2-KO/Wt-oocyte) >2 or <0.5). [file 1756-8935-2-9-S1.pdf]

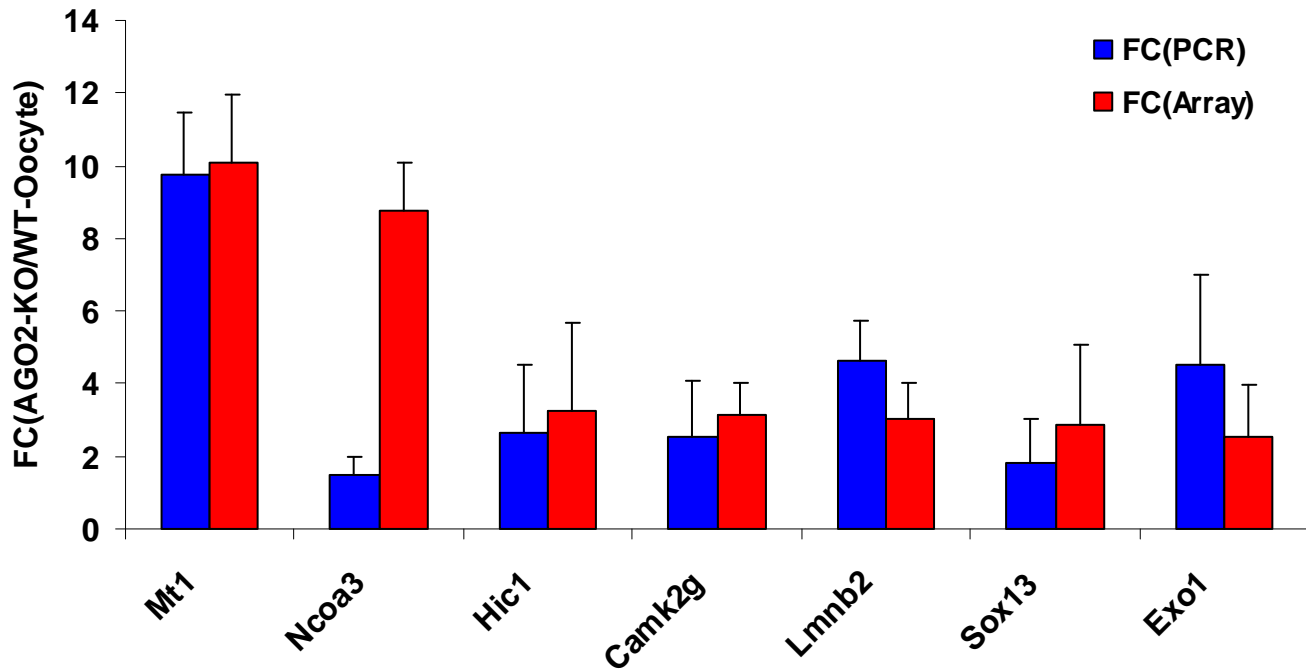

Supplement: Additional file 2 — Real-time PCR measurement of candidate gene expression. Seven genes showing differential expression based on array results were checked by single cell cDNA real-time PCR. Six of them were confirmed as showing differential expression. [file 1756-8935-2-9-S2.pdf]
